# Supplementary material for: NetAct: a computational platform to construct core transcription factor regulatory networks using gene activity
Source: Genome Biol. 2022 Dec 27;23:270. doi: 10.1186/s13059-022-02835-3 (PMC9793520; doi:10.1186/s13059-022-02835-3)
Supplement: Supplementary file 4 — Additional file 4: Review history. [file 13059_2022_2835_MOESM4_ESM.docx]

Review History

**First round of review**

**Reviewer 1**

**Were you able to assess all statistics in the manuscript, including the appropriateness of statistical tests used?**

Yes: No advanced statistics methods were used.

**Were you able to directly test the methods?**

No.

**Comments to author:**

This manuscript presents a new computational platform for constructing core transcription-factor regulatory networks, named NetAct. NetAct uses transcriptomics data and literature-based transcription factor-target information. NetAct contains methodology to infer the activities of regulators using the expression of target genes, methodology to constructs networks of transcription factor, and methodology to describe the dynamics of activation of these networks using the RACIPE framework previously developed by the authors. The manuscript includes comparisons with other relevant software and describes two case studies. The integration of network construction/ inference with dynamic modeling is a valuable advance. Unfortunately, the manuscript is unclear on multiple points, thus it is difficult to evaluate its technical merit.

Major comments

1. The working hypotheses that underlie the method of assigning an activity level to each transcription factor are insufficiently justified and validated. One hypothesis is that each transcription factor's targets can be grouped into two groups of co-expressed genes. A specific community identification method is used to detect the two groups. The validity of the results should be checked by (i) using the modularity measure Q introduced by Mark Newman (the same author whose clustering method is used here) and (ii) using other methods.

2. Related comment: Line 181 "Genes within the red block are positively correlated with each other, while genes from the blue blocks are negatively correlated with each other." Isn't it rather the case the blue blocks contain negative correlations and are a sign that the genes in the two red blocks are anti-correlated with each other?

3. In the method to assign activity to a transcription factor the rationale of subtracting the expression of target genes inhibited by the TF is not given. Inhibiting target genes is also a form of activity.

4. More information needs to be added about RACIPE than "..takes network topology as the input and generates an ensemble of mathematical models with random kinetic parameters." What are the variables of does RACIPE (e.g., are there separate variables for mRNA and protein)? What are the functional forms of the interactions? Mass action kinetics may not be well suited to transcriptional regulation. What are the assumed bounds on the values of kinetic parameters? An example equation would also be very informative.

5. Epithelial to mesenchymal transition in response to TGFb was studied by previous models, for example reference 19 in the manuscript. The results of the current analysis should be compared to this relevant prior work.

More minor comments

Lines 68-81 describe an approach to integrate top-down and bottom-up approaches to infer and model core gene regulatory networks. There has been a follow-up to reference 27 cited in this paragraph, which is relevant to this work because it incorporates activity of transcription factors: https://doi.org/10.3390/jpm11030193

Line 139-142. The databases used need a more informative description, to help the reader understand which of the transcription factor - target gene interactions are observed and which are inferred.

Line 152 "to evaluate whether the enrichment analysis can detect the perturbed TFs". This was not clear at first reading. Specifically, it was not clear how it is possible to have a negative answer. After reading the whole manuscript, including the SI, I have a better understanding, but this will not be the case for a novice reader. The paragraph should start with a brief introduction to GSEA, then explain what the task is, and why it is not obvious that the perturbed TF would be detected.

Line 300. "We found that, consistent with the expression and activity profiles, the network clearly allows two distinct transcriptional clusters that can be associated with E and M states". This sentence needs to be supported by evidence, e.g. by referring to specific panels of Figure 5.

Lines 328 More elaboration is needed to support the sentence "The expression and activity profiles of these TFs captures the essential dynamics of transcriptional state transitions during macrophage polarization (Fig 6abc). " What is the essential dynamics of transcriptional state transitions? In what way do the static expression/activity profiles reflect these dynamics?

Line 437 "k-mean clustering is applied". What is the value of k? Is it 2?

Line 478 indicates that TF-TF interactions are filtered out if they are not in the transcription factor- target database. Which specific database is this? Is it possible that signaling-type interactions are not included in this database?

In Figure 2f,g how is percentage defined? How can the positive percentage and negative percentage both be 100%?

Figure 3 gives "target activity" as one of the variables. Can a target activity be defined for anything else than transcription factors? If not, there must be significantly fewer datapoints that inform the histogram that has target activity than the histogram that has target expression. Then how come some of the histograms are very similar?

Figure 5: it seems that some references to panels of this figure are incorrect. What do the colors mean in panel d? The figure caption does not describe panel e. What does the "color key and histogram" mean? ( i.e., Which panels use this color key? Histogram of what?).

Figure 6. What specific evidence supports the conclusion that the results in panel f match those of panel a? What does the "color key and histogram" mean?

**Reviewer 2**

**Were you able to assess all statistics in the manuscript, including the appropriateness of statistical tests used?**

Yes: All statistics in the manuscript is reasonable according to the presentation.

**Were you able to directly test the methods?**

No.

**Comments to author:**

Su et al propose a computational platform named NetAct to construct core transcription-factor regulatory networks with transcriptomics data and literature-based TF-target databases. The manuscript evaluated the performance of NetAct on both simulated datasets and real datasets. I still have some major concerns about the manuscript, mainly regarding the methodology and comparisons with existing tools. Please see my detailed comments below.

Major issues

1. In eq.1, the I(i) is set to 1 or -1 based on the active or repressed group. How accurate are these targets? For example, are all targets in one group activated and all targets in the other group inhibited by this TF? Also, do the scores of active targets counteract the scores of repressive targets when calculating the TF activities?

2. Why does the "n is set to 1/5" have the best performance in eq.2? The authors should at least show some evidence to support the parameter definition.

3. In the inferring TF activities, the TF-target pairs are filtered from the database. What if the user wants to find novel TF-target pairs? Is that possible that NetAct can find novel TF-targets pairs? This is important as most of the TF targets could be unknown in real scenarios.

4. How are the literature-based TF-targets databases used in NetAct for the simulation analyses in Fig 3 and Fig 4? The synthetic TF network should be much different from known databases. Did the authors use the TF-target pairs in the simulated network as the ground truth?

5. There are many different GRN inference methods, which have been systematically reviewed in Pratapa et al., Nature Methods 2020. The authors only compared the performance of NetAct with NCA, VIPER, and AUCell, what is the performance of NetAct compared to the methods that showed better performance in the above evaluation paper, such as PIDC, GENIE3, and GRNBOOST2?

Minor issues

1. On page 7 line 159, the authors mentioned that "Furthermore, these corresponding q-values from all the gene expression data are converted to specificity and sensitivity values (see Methods)". However, there is no further information in the Methods part. The authors should address this.

2. Fig 2a-c does not have figure legends.

3. The texts in Fig 5 and Fig 6 are fuzzy.

**Authors Response**

**Point-by-point responses to the reviewers’ comments:**

Reviewer #1:
This manuscript presents a new computational platform for constructing core transcription-factor regulatory networks, named NetAct. NetAct uses transcriptomics data and literature-based transcription factor-target information. NetAct contains methodology to infer the activities of regulators using the expression of target genes, methodology to constructs networks of transcription factor, and methodology to describe the dynamics of activation of these networks using the RACIPE framework previously developed by the authors.  The manuscript includes comparisons with other relevant software and describes two case studies. The integration of network construction/ inference with dynamic modeling is a valuable advance. Unfortunately, the manuscript is unclear on multiple points, thus it is difficult to evaluate its technical merit.

Major comments

1. The working hypotheses that underlie the method of assigning an activity level to each transcription factor are insufficiently justified and validated. One hypothesis is that each transcription factor's targets can be grouped into two groups of co-expressed genes. A specific community identification method is used to detect the two groups. The validity of the results should be checked by (i) using the modularity measure Q introduced by Mark Newman (the same author whose clustering method is used here) and (ii) using other methods.

*Reply: We thank the reviewer for the suggestion of evaluating other modularity methods. In NetAct, we implement the same community detection algorithm from Mark Newman’s Method [1]. But in addition, we further filter out less informative genes in the detected communities corresponding to the activation or inhibition directions. See details in Inferring TF activity in Methods Section. To validate the communities, we have conducted the following new analysis by using the experimental data set (GEO: GSE17172) as follows:*

*1. For the co-expression matrix of the target genes from a certain TF, we used another two established community detection algorithms including greedy optimization [2,3] and leading eigenvector [4] for finding communities.*

*2. For each detected community, we calculate the overlapping similarity with two set of communities corresponding to activation or inhibition from NetAct approach. The similarity is measured by* $\max{|D}_{i}\cap C_{+/-}|/|C_{+/-}|$*. Here,* $C_{+/-}$ *denotes the community from either the activation or inhibition group by NetAct, and* $D_{i}$ *denotes the* $i^{th}$ *community detected by other algorithms (left panel for greedy optimization and right panel for leading eigenvector). Our results show that the gene members from one community by NetAct approach largely overlap with the communities from other approaches.*

*Moreover, the activity inference algorithm has been extensively benchmarked with our in-silico simulated data sets, as shown in* ***Figs. 3*** *and* ***4****.*

*We have included the figure as Fig.S1.*

**Fig. S1: Comparison of the grouping schemes by Newman’s method (NetAct) and other community detection algorithms.** Two other community detection algorithms were applied to divide target genes of enriched transcription factors into two groups using the gene expression data from shRNA knockdown of FOXM1 in lymphoma cells (GEO: GSE17172). Here, we applied two other community detection algorithms: greedy optimization (left) and leading eigenvector (right) algorithms. Within the detected communities, we calculated the maximum percentage of genes overlapped with the gene members detected by NetAct (defined as similarity). As shown, the gene members from one community corresponding to the +/- regulatory direction can be largely overlapped with other grouping schemes from other community detection algorithms.

*We have also added the following sentences in the main text.*

*We also explored a few other community detection algorithms^78–80^ and found they produced similar results (Fig. S1).*

*Reference:*

*1. Newman MEJ. Modularity and community structure in networks. Proc Natl Acad Sci U S A. 2006;103:8577–82.*

*2. Reichardt J, Bornholdt S. Statistical mechanics of community detection. Phys Rev E. American Physical Society; 2006;74:016110.*

*3. Newman MEJ. Analysis of weighted networks. Phys Rev E. American Physical Society; 2004;70:056131.*

*4. Newman MEJ. Finding community structure in networks using the eigenvectors of matrices. Phys Rev E. 2006;74:036104.*

2. Related comment: Line 181 "Genes within the red block are positively correlated with each other, while genes from the blue blocks are negatively correlated with each other." Isn't it rather the case the blue blocks contain negative correlations and are a sign that the genes in the two red blocks are anti-correlated with each other?

*Reply: We thank the referee to point out the confusion in the sentence. To clarify it, we have revised the sentence as follows.*

*Within the red blocks, the expression of any column gene is positively correlated with that of any row gene; while within the blue blocks, the expression of any column gene is negatively correlated with that of any row gene. This indicates that the genes in the two red blocks are anti-correlated in gene expression with each other.*

3. In the method to assign activity to a transcription factor the rationale of subtracting the expression of target genes inhibited by the TF is not given. Inhibiting target genes is also a form of activity.

*Reply: We thank both the referees for asking this question. The rationale to subtract the expression of target genes from the two groups is that their gene expressions are anti-correlated. Subtracting actually reinforces the inference of TF activity. The higher the TF activity, the more the inhibitory targets are suppressed. Moreover, our in-silico simulations and benchmark indicate the effectiveness of this definition of TF activity. To clarify this point more, we have added the following explanation in page 19:*

*For genes in the inhibition group, the higher the TF activity, the more the genes are suppressed. Thus, the formula in Equation (1) captures well the activity of TFs for their effects to both activating and inhibitory targets.*

4. More information needs to be added about RACIPE than "..takes network topology as the input and generates an ensemble of mathematical models with random kinetic parameters." What are the variables of does RACIPE (e.g., are there separate variables for mRNA and protein)? What are the functional forms of the interactions? Mass action kinetics may not be well suited to transcriptional regulation. What are the assumed bounds on the values of kinetic parameters? An example equation would also be very informative.

*Reply: To address this issue, we have provided a lot more details of the RACIPE method in “SI5. Simulation of activity and expression using RACIPE”. In the revision, we provided the details of the mathematical equations to simulate both gene expression and activity, the description of the kinetic parameters, and the choice of their boundary.*

*(The changes are not provided here as it is long. Please see the revised Supplemental Information SI5)*

5. Epithelial to mesenchymal transition in response to TGFb was studied by previous models, for example reference 19 in the manuscript. The results of the current analysis should be compared to this relevant prior work.

*Reply: We have added new text to compare the network modeling results with the EMT network model by reference 19 as follows.*

*Compared to a previous model of the EMT network based on an extensive literature survey, the GRN constructed by NetAct identified some of the same regulators induced by the TGF-β pathway, such as SMAD3/4, TWIST2, ZEB1, CTNNB1, NFKB1, RELA, FOS and EGR1. Because of the lack of microRNAs and protein-protein interactions in the database, NetAct didn’t identify factors like miR200 and signaling molecules like PI3K. Interestingly, the NetAct model identifies STAT1/3, which was connected to other signaling pathways, such as HGF, PDGF, IGF1and FGR, but not TGF-β in the previous network model. In addition, the NetAct model identified regulators in other important pathways in TGF-β-induced EMT in cancer cells, e.g., cell cycle pathway (RB1 and E2F1) and DNA damage pathway (P53).*

More minor comments

Lines 68-81 describe an approach to integrate top-down and bottom-up approaches to infer and model core gene regulatory networks. There has been a follow-up to reference 27 cited in this paragraph, which is relevant to this work because it incorporates activity of transcription factors: [https://nam12.safelinks.protection.outlook.com/?url=https%3A%2F%2Fdoi.org%2F10.3390%2Fjpm11030193&amp;data=05%7C01%7Ca.katebi%40northeastern.edu%7C83af582f9feb499d8d7e08da5c240f9d%7Ca8eec281aaa34daeac9b9a398b9215e7%7C0%7C0%7C637923607203253603%7CUnknown%7CTWFpbGZsb3d8eyJWIjoiMC4wLjAwMDAiLCJQIjoiV2luMzIiLCJBTiI6Ik1haWwiLCJXVCI6Mn0%3D%7C3000%7C%7C%7C&amp;sdata=ejUS9CiZFnkmIUcHcqbUK6wjhPnm9QuxKhEkZ6YtCTQ%3D&amp;reserved=0](https://nam12.safelinks.protection.outlook.com/?url=https%3A%2F%2Fdoi.org%2F10.3390%2Fjpm11030193&data=05%7C01%7Cm.lu%40northeastern.edu%7C42ad1ba4def442a2958108da5c5f5557%7Ca8eec281aaa34daeac9b9a398b9215e7%7C0%7C0%7C637923861782083731%7CUnknown%7CTWFpbGZsb3d8eyJWIjoiMC4wLjAwMDAiLCJQIjoiV2luMzIiLCJBTiI6Ik1haWwiLCJXVCI6Mn0%3D%7C3000%7C%7C%7C&sdata=%2FfaQvbzYjiEcde7cPfO9vL7kad25BKf9M63z1jlEeW8%3D&reserved=0)

*Reply: We thank the referee for the suggestion. We have added the reference.

Line 139-142. The databases used need a more informative description, to help the reader understand which of the transcription factor - target gene interactions are observed and which are inferred.*

*We have provided details of the databases in Table S1 and Supplemental Information SI1. We have also cited them in the lines mentioned above.*

Line 152 "to evaluate whether the enrichment analysis can detect the perturbed TFs".  This was not clear at first reading. Specifically, it was not clear how it is possible to have a negative answer. After reading the whole manuscript, including the SI, I have a better understanding, but this will not be the case for a novice reader. The paragraph should start with a brief introduction to GSEA, then explain what the task is, and why it is not obvious that the perturbed TF would be detected.

*Reply: We thank the referee for the suggestion. We have added the following text at the beginning of the paragraph.*

*GSEA is a popular statistical method that can be used to evaluate significant overlapping between a set of genes and differentially expressed genes between two experimental conditions. Using various types of TF-target databases, our goal is to find the best version of the database, so that GSEA can detect the target gene sets of the relevant TFs to be statistically significant.*

Line 300. "We found that, consistent with the expression and activity profiles, the network clearly allows two distinct transcriptional clusters that can be associated with E and M states". This sentence needs to be supported by evidence, e.g. by referring to specific panels of Figure 5. 

*Reply: We have cited the specific panels of Figure 5 in this sentence.*

*We found that, consistent with the expression and activity profiles (Fig. 5a), the network clearly allows two distinct transcriptional clusters that can be associated with E (the yellow cluster in Fig. 5d) and M states (the blue cluster in Fig. 5d).*

Lines 328 More elaboration is needed to support the sentence "The expression and activity profiles of these TFs captures the essential dynamics of transcriptional state transitions during macrophage polarization (Fig 6abc). " What is the essential dynamics of transcriptional state transitions? In what way do the static expression/activity profiles reflect these dynamics?

*Reply: We actually explained the findings from the expression and activity profiles in the next few sentences. To clarify the logical flow of this part of results, we have modified the sentence to:*

*The expression and activity profiles of these TFs (Fig. 6abc) capture the essential dynamics of transcriptional state transitions during macrophage polarization as follows.*

Line 437 "k-mean clustering is applied". What is the value of k? Is it 2?

*Reply: We use k = 1. K-mean clustering was applied to find the center vector of gene expression correlation. We have added (k = 1) in the following sentence.*

*(2) k-mean algorithm (k = 1) is performed within each cluster to determine the center vector.*

Line 478 indicates that TF-TF interactions are filtered out if they are not in the transcription factor- target database. Which specific database is this? Is it possible that signaling-type interactions are not included in this database?

*Reply: The TF-target database is D1 compiled in NetAct (see Table S1). It is indeed likely that many signaling interactions are not presented. It could be a good idea to use a broader TF interaction database for this filtering step in future work. We have added more details to this sentence.*

*Interactions are filtered out if they cannot be found in the TF-target regulatory database (i.e., D1).*

In Figure 2f,g how is percentage defined? How can the positive percentage and negative percentage both be 100%?

*Reply: The figure caption for Fig.2fg was confusing. We have expanded the caption to clarify the definition.*

*(****f****,* ***g****) show the proportion of genes from the activation group that are positively correlated with the TF expression (red bars) and the proportion of genes from the inhibition group that are negatively correlated with the TF expression (blue bars).*

Figure 3 gives "target activity" as one of the variables. Can a target activity be defined for anything else than transcription factors? If not, there must be significantly fewer datapoints that inform the histogram that has target activity than the histogram that has target expression. Then how come some of the histograms are very similar?

*Reply: The referee is right that we defined “target activity” the same as target expression for non-TF genes in this calculation. That is why the histograms look similar. There was also an error in the label of the y-axis. They should be normalized density, not counts. We have fixed the figure and explained the target activity in the figure caption.*

Figure 5: it seems that some references to panels of this figure are incorrect. What do the colors mean in panel d? The figure caption does not describe panel e. What does the "color key and histogram" mean? ( i.e., Which panels use this color key? Histogram of what?).

*Reply: We thank the referee for pointing out these issues. The description of the color bars and the color legend for Fig.5d, and the caption for Fig.5e has been included in the revision. The color legend is for Fig.5d. Moreover, the wrong references to Fig.5 panels have been fixed. In the caption of Fig.5, we have added:*

*The blue cluster represents the mesenchymal state and the yellow cluster represents the epithelial state. The color legend for the heatmap is at the bottom right.* ***(e)*** *Knockdown simulations of the TF regulatory network. The bar plot shows the proportion of RACIPE models in each state (epithelial or mesenchymal) for the conditions of the knockdown of every TF.*

Figure 6. What specific evidence supports the conclusion that the results in panel f match those of panel a? What does the "color key and histogram" mean?

*Reply: The “color key and histogram” panels in both Figs 5 and 6 are for the heatmap of the RACIPE simulated data. The histogram shows the distribution of all the simulated data, and the colors illustrate the gene expression/activity levels in the heatmap.*

*In the SI7, we described how RACIPE models can be mapped to the experimental data.*

*Each resulting model was mapped back to experimental states by first averaging the experimental values of the simulated genes in each experimental condition and then identifying the experimental condition with the lowest Euclidean distance to each model.*

*We have also added the following in the SI7.*

*The color schemes of the mapping can be observed in Fig. 6f for the simulated heatmap and in Fig. 6e for the data projected to the first two PCs. The structure of the projected simulation data* *is very close to the structure of the projected experimental data. Moreover, we observed consistent patterns of gene expression/activity in the experimental and simulation data. For example, we found high Sp1/3 and Foxo3 in the UT condition but low in other conditions, high Stat1 and Nfkb1 in the IFNγ and IFNγ +IL4 conditions, high Rb1 and Smad1 in the UT and IL4 conditions, and high Myc and Ep300 in the IL4 condition. These observations are largely consistent with the literature^34^.*

Reviewer #2: Su et al propose a computational platform named NetAct to construct core transcription-factor regulatory networks with transcriptomics data and literature-based TF-target databases. The manuscript evaluated the performance of NetAct on both simulated datasets and real datasets. I still have some major concerns about the manuscript, mainly regarding the methodology and comparisons with existing tools. Please see my detailed comments below.

Major issues

1. In eq.1, the I(i) is set to 1 or -1 based on the active or repressed group. How accurate are these targets? For example, are all targets in one group activated and all targets in the other group inhibited by this TF? Also, do the scores of active targets counteract the scores of repressive targets when calculating the TF activities?

*Reply: We thank both the referees for asking this question. The rationale to subtract the expression of target genes from the two groups is that their gene expressions are anti-correlated. Subtracting actually reinforces the inference of TF activity. The higher the TF activity, the more the inhibitory targets are suppressed. Moreover, our in-silico simulations and benchmark indicate the effectiveness of this definition of TF activity.*

*To address the first two questions, we looked into the data of our in-silico benchmark analysis and calculated the accuracy of the target grouping according to the ground-truth regulatory interactions. As shown in the new Fig. S9, we found that almost all targets in one group are activated by the TF, and almost all targets in the other group are inhibited by the TF when using the original regulon database. But the accuracy drops when the regulon database is perturbed.*

**Fig. S9. Comparison of target genes’ grouping scheme with the synthetic gene regulatory network.** In this test, simulated gene expression and regulon databases of different perturbation levels were provided to NetAct to find the grouping scheme for the targets of each TF. For targets in the ground-truth network, we evaluated the accuracy of the grouping scheme, *i.e.*, whether NetAct correctly predicts the activation/inhibition of the targets by the TF. Y-axis shows the accuracy defined as the fraction of TF-target pairs from NetAct whose activation/inhibition nature is consistent with the ground-truth network. X-axis shows the perturbation levels of the benchmark data.

*Reply: We have included the figure as Fig. S9 and the following explanation in page 19:*

*According to our in-silico benchmark test (Fig. S9), we found that majority of the targets in one group are activated by the TF, and majority of those in the other group are inhibited by the TF. For genes in the inhibition group, the higher the TF activity, the more the genes are suppressed. Thus, the formula in Equation (1) captures well the activity of TFs for their effects to both activating and inhibitory targets.*

2. Why does the "n is set to 1/5" have the best performance in eq.2? The authors should at least show some evidence to support the parameter definition.

*Reply: We thank the reviewers for pointing out the issue. In the revision, we add the results for the analysis of the n optimization. In short, we optimize* $n$ *in the in-silico benchmark to minimize the difference between ground truth TF activity and inferred TF activity. The data set is the same as those used in the in-silico TF activity benchmark for the untreated condition (****Fig. 3****). For TF activity inference, we use a synthetic TF-target regulatory database (explained below) and the simulated expression of target genes. We evaluate the performance of the TF activity inference by applying NetAct 101 times with different TF-target databases, one derived from the original TF-target relationship and the other 100 derived from the database with 25% perturbations (same as those used in* ***Figs. 3*** *and* ***4****). For each case, we infer the TF activities using NetAct with different n values from the Hill function. Then, we compute the pairwise correlation between the simulated activities and true activities for all TFs and report the average Spearman correlation as the metric for accuracy. As shown in the following trace plot, when using n from* $\frac{1}{12}$ *to* $\frac{1}{4}$*, there is no apparent difference in terms of overall performance. But when choosing* $n$ *from* $\frac{1}{2}$ *to 4, the mean correlation decreases dramatically. We thus choose 1/5 for n in NetAct. Based on the new data, the choice of n is quite flexible, as long as it is small enough (meaning in favoring of the weights for DE genes).*

*We have included the figure as* ***Fig. S8****.*

**Fig.S8: Optimization of the Hill coefficient in the TF activity inference**. We used in-silico benchmark dataset together with the synthetic TF regulatory datasets to optimize the Hill coefficient $n$ in the Hill function for inferring the TF activity. From a series of different Hill coefficients, we measured the performance by reporting the spearman correlation between the inferred activities and ground-true activities. We found that when $n$ is small enough ($<\frac{1}{4}$), the performance remains comparably good.

3. In the inferring TF activities, the TF-target pairs are filtered from the database. What if the user wants to find novel TF-target pairs? Is that possible that NetAct can find novel TF-targets pairs? This is important as most of the TF targets could be unknown in real scenarios.

*Reply: In the current study, we emphasize on the literature-based TF-target relationships to ensure the quality of regulatory interactions. To evaluate the capability of NetAct in finding novel TF-target pairs, we used the in-silico data to check the performance of NetAct in finding the ground-truth interactions not presented in the regulon database for the cases when the regulon database is perturbed by 25%, 50% or 75%. More details of the benchmark analysis for network construction are provided below in point 5.*

4. How are the literature-based TF-targets databases used in NetAct for the simulation analyses in Fig 3 and Fig 4? The synthetic TF network should be much different from known databases. Did the authors use the TF-target pairs in the simulated network as the ground truth?

*Reply: In the benchmark test, the TF-target database was constructed from the synthetic network, not the literature-based regulatory database. So yes, in the new network construction benchmark, we used the TF-target relationships in the synthetic network as the ground truth. But in this benchmark, we also perturbed the TF-target database so that a large portion of ground truth interactions are not provided in the TF-target database. See below for more details.*

5. There are many different GRN inference methods, which have been systematically reviewed in Pratapa et al., Nature Methods 2020. The authors only compared the performance of NetAct with NCA, VIPER, and AUCell, what is the performance of NetAct compared to the methods that showed better performance in the above evaluation paper, such as PIDC, GENIE3, and GRNBOOST2?

*Reply: In the revision, we used the in-silico simulation data set and performed a network construction benchmark of NetAct and a few other network construction algorithms, as shown in the new figures* ***Fig. 4bcd****. NetAct, using the TF activity inferred from the original regulon database, outperforms not only network construction methods using gene expression, such as GENIE3, GRNBoost2, and ppcor, but also GENIE3 using the TF activity inferred by AUCell (****Fig. 4b****). The last approach was presented to mimic a popular method SCENIC. Moreover, we evaluated the performance of NetAct when using a perturbed regulon database. We found that NetAct remains performing well when the perturbation level is as large as 50%, when evaluated by all the ground-truth interactions (****Fig. 4c****) and by those not presented in the regulon database (****Fig. 4d****). The latter case was designed to evaluate the capability of NetAct in predicting novel interactions.*

*We have provided the detailed benchmark methods in the new SI6 and the results in Fig.4 and Fig.S7. We also described the above results in the main text.*

**Fig. 4. (b-d)** Network inference. The panels show the performance of network inference algorithms from the simulation benchmark by the precision and recall for different link selection thresholds. **(b)** Network inference performance against all ground-truth regulatory interactions. Tested methods are GENIE3, GRNBoost2, and PPCOR, using transcription factor (TF) expression; GENIE3 using TF activity inferred by AUCell; NetAct using its inferred TF activity. For the latter two methods, original (unperturbed) regulons obtained from the regulatory network were used. **(c)** Network inference performance of NetAct against all ground-truth regulatory interactions using the regulons with 0% (the original), 25%, 50%, and 75% target perturbations. **(d)** Network inference performance of NetAct in discovering new regulatory interactions not existing in the regulons. NetAct was applied using the regulons at different perturbation levels (25%, 50%, and 75%). The benchmark results shown here are for the case of the untreated simulation. The results for the case of the knockdown simulation are shown in **Fig. S7**.

Minor issues

1. On page 7 line 159, the authors mentioned that "Furthermore, these corresponding q-values from all the gene expression data are converted to specificity and sensitivity values (see Methods)". However, there is no further information in the Methods part. The authors should address this.

*Reply: We have provided the description for the AUC calculations in Methods.*

*In the database benchmark test, for each database, we computed the sensitivity and specificity values for different q-value cutoffs. Here, for each cutoff value, we defined the sensitivity as the proportion of data sets where the gene sets for the KD TFs were enriched with q-values below the cutoff value. We also defined specificity as the fraction of cases where the gene sets for the other TFs (non-KD TFs in the benchmark) were not enriched with q-values above the cutoff value. We then computed area under the ROC curve (AUC) using the DescTools R package^76^.*

2. Fig 2a-c does not have figure legends.

We have provided the figure legends for those panels.

3. The texts in Fig 5 and Fig 6 are fuzzy.

*Reply: We have provided the high-resolution version of the figures.*

**Second round of review**

**Reviewer 2**

The revised manuscript has explained the rationales of parameter settings and added the comparison with other methods. I think the manuscript is now in a much better condition.

Here are my points on the authors’ response to Reviewer#1.

Multiple concerns concerning the manuscript were noted in reviewer #1's comments.

1. The reviewer requested the authors fully justify and validate the underlying working hypotheses of the proposed method. The authors used two more community detection approaches to assess this and discovered that the results were similar.

2. The reviewer asked the author to explain the rationale for subtracting the expression of target genes inhibited by the TF. The authors clarified that subtraction is effective in defining the TF activity.

3. The reviewer requested that the author compare their EMT results to relevant earlier research. The author updated the references and included the comparison results.

The rest suggestions made by the reviewer include the details that the prior draft omitted. Point by point, the authors offered reasonable responses.
